# Minimally Invasive Techniques for Managing Dental Caries in Children: Efficacy, Applications, and Future Directions

**DOI:** 10.7759/cureus.87450

**Published:** 2025-07-07

**Authors:** Arwa A AL-Kaff, Abdulrahman Z Alshehri, Rana A Alasmari, Najd Alsubaie, Albandari Aldaws, Ameera Althaqeel, Rola S Alshehri, Yasmin M Alawaji

**Affiliations:** 1 Pediatric Dentistry, Ministry of Health, Riyadh First Health Cluster, Riyadh, SAU; 2 Dentistry, College of Dentistry, King Saud University, Riyadh, SAU; 3 Dentistry, Princess Nourah bint Abdulrahman University, Riyadh, SAU; 4 Dentistry, Princess Nourah Bint Abdulrahman University, Riyadh , SAU; 5 Dentistry, Riyadh Elm University, Riyadh , SAU; 6 General Dentistry, Princess Nourah bint Abdulrahman University, Riyadh, SAU

**Keywords:** atraumatic restorative treatment, bioactive materials, caries management, hall technique, minimally invasive dentistry, pediatric dentistry, resin infiltration, silver diamine fluoride (sdf), teledentistry

## Abstract

Minimally Invasive Dentistry (MID) represents a transformative approach in pediatric caries management, emphasizing tooth preservation, patient comfort, and preventive care over conventional invasive methods. The aim of this narrative review is to evaluate the efficacy, clinical applications, and future directions of various MID techniques in children, including silver diamine fluoride (SDF), atraumatic restorative treatment (ART), the Hall Technique, resin infiltration, and bioactive restorative materials. These methods have shown considerable success in reducing treatment complexity and improving outcomes, especially for high-risk and special healthcare needs (SHCN) populations. A structured literature search was conducted across PubMed, Scopus, and Google Scholar databases for studies published between 2000 and 2025. Search terms included “minimally invasive dentistry in children,” “silver diamine fluoride pediatric caries,” “atraumatic restorative treatment,” and related techniques. Inclusion criteria encompassed clinical trials, systematic reviews, and expert consensus articles, while animal studies and those lacking rigorous methodology were excluded. Results indicate that MID techniques such as SDF can arrest caries in 80-90% of cases, while ART and the Hall Technique provide minimally invasive, cost-effective alternatives to conventional restorations. Resin infiltration improves esthetics and arrests lesion progression in non-cavitated teeth, while bioactive materials promote remineralization and pulp preservation. Emerging technologies, including laser-assisted caries removal, air abrasion, and AI-driven diagnostics, further enhance MID accessibility and precision. Despite promising outcomes, challenges such as clinician training deficits, parental concerns, and lack of long-term evidence persist. Future efforts should focus on addressing these barriers through research, education, and policy reform to facilitate the integration of MID into routine pediatric dental practice.

## Introduction and background

Dental caries is the most prevalent non-communicable disease in children, affecting more than 600 million school-age children worldwide and disproportionately burdening underprivileged and underdeveloped regions [1-3]. "Early Childhood Caries (ECC) is defined as the presence of one or more decayed, non-cavitated or cavitated lesions, missing or filled due to caries surfaces, in any primary tooth of a child under six years of age [4]. ECC frequently results in systemic health issues such as malnourishment, pain, decreased quality of life, and developmental delays, as well as an increased risk of new caries lesions in both the primary and permanent dentitions [3,4]. Conventional restorative approaches, such as invasive drilling and amalgam fillings, often exacerbate dental anxiety in pediatric patients, contribute to unnecessary removal of healthy tooth structure, and remain inaccessible in resource-limited settings. Conventional methods for the restoration of dental caries may cause distress in patients. To lower the levels of anxiety experienced by children, minimal intervention dentistry principles can be used to minimize dental anxiety [5]. These challenges have driven the widespread adoption of minimally invasive dentistry, a patient-centered philosophy that prioritizes early caries detection, prevention, and tooth preservation through biologically friendly techniques [6]. 

Minimally invasive dentistry strategies such as silver diamine fluoride, atraumatic restorative treatment, the Hall Technique, fluoride varnishes, and bioactive materials are transforming pediatric caries management. For instance, SDF a non-invasive, cost-effective topical agent has been shown to arrest caries progression in primary teeth by up to 80%, making it particularly valuable for underserved populations and children with dental anxiety or special healthcare needs [7,8]. ART, which utilizes hand instruments and glass ionomer cement, eliminates the need for local anesthesia and electrical equipment, reducing treatment costs and improving access in low-resource settings [9]. Regular application of fluoride varnish, administered two to four times per year, has been shown to effectively lower the incidence of dental caries, reducing carious surfaces by 43% in permanent teeth and 37% in primary teeth [10]. 

Despite these advancements, barriers to MID adoption persist. Parental concerns about SDF’s aesthetic effects (e.g., irreversible black staining), inconsistencies in clinician training, and limited long-term data on novel bioactive materials hinder widespread implementation [11,12]. Emerging technologies, such as laser-assisted caries removal, air abrasion, and teledentistry, offer promising solutions but require rigorous evaluation to ensure feasibility and scalability in diverse pediatric populations [13,14].

This comprehensive review synthesizes current evidence on minimally invasive techniques for managing dental caries in children, evaluates their clinical efficacy and practical applications, and explores future directions for research and innovation. By addressing knowledge gaps and advocating for evidence-based MID protocols, this review aims to inform clinical practice, policymaking, and global strategies to reduce the burden of childhood caries.

## Review

Materials and methods

A narrative literature search was conducted using PubMed, Scopus, and Google Scholar to identify relevant studies published between 2000 and 2025 on minimally invasive techniques for managing dental caries in children. The search terms included "minimally invasive dentistry in children," "silver diamine fluoride for pediatric caries," "atraumatic restorative treatment in children," and "Hall technique for primary molars." The search was restricted to articles written in English that focused on human participants. To ensure a comprehensive review, manual searches of reference lists from selected studies were performed to identify additional relevant literature. The inclusion criteria encompassed clinical trials, systematic reviews, meta-analyses, and expert consensus statements evaluating the effectiveness, clinical outcomes, and long-term success of minimally invasive dentistry techniques in pediatric populations, including silver diamine fluoride, atraumatic restorative treatment, the Hall Technique, fluoride varnish, and pit and fissure sealants. Studies investigating the application of MID approaches in children with special healthcare needs (SHCN) were also included. Exclusion criteria were established to omit studies focusing on adult populations, case reports, editorials, opinion pieces without clinical evidence, animal studies, and research lacking clear methodology, outcome measures, or sufficient follow-up data.

Results and discussion

*Minimally Invasive Preventive Strategies* 

Risk assessment and personalized prevention plans: Evaluating a child's caries risk is essential for formulating targeted preventive strategies that correspond to their specific risk factors, thereby promoting a personalized approach to oral healthcare [15]. The Caries Risk Assessment (CRA) instrument enables dental practitioners to classify patients into low, moderate, and high-risk categories. This classification enables prompt interventions via customized preventive strategies [15]. Caries Management by Risk Assessment (CAMBRA) is a notable risk assessment model in pediatric dentistry. This methodology integrates clinical evaluations, dietary assessments, and fluoride exposure analyses to guide appropriate preventive and restorative interventions [15]. CAMBRA-based strategies involve the use of specific fluoride varnishes, the application of sealants, dietary guidance, and modifications to recall intervals, all tailored to the individual risk profile of each patient [15]. Studies demonstrate that interventions grounded in caries risk assessment can decrease caries incidence by 40-60% in children over five years classified as high-risk [16]. Furthermore, the incorporation of CRA into dental curricula in educational institutions and public health environments has proven effective in reducing the incidence of early childhood caries, particularly in communities with limited access to routine dental care [16]. The advancement of artificial intelligence (AI)-based clinical risk assessment tools offers substantial prospects for enhancing diagnostic accuracy and the early detection of high-risk patients [17]. Artificial intelligence-driven algorithms can analyze clinical, dietary, and behavioral data, providing prompt, evidence-based recommendations for personalized preventive care [17].

Preventive strategies, including dietary modifications, oral hygiene education, and personalized caries risk assessments, are crucial for the effective management of dental caries in children. Evidence-based strategies, such as reducing sugar intake, promoting fluoride toothpaste, and implementing oral health programs in schools, have demonstrated significant efficacy in lowering caries prevalence [16,17]. Moreover, personalized risk assessment models like CAMBRA enable targeted preventive strategies, leading to improved long-term oral health outcomes [18,19].

Fluoride varnishes: Fluoride varnish is a concentrated fluoride formulation applied by professionals to prevent dental caries and enhance enamel remineralization [10]. The mechanism of action entails the creation of a calcium fluoride-like deposit on the enamel surface, serving as a reservoir for fluoride ions. These ions are gradually released over time, facilitating remineralization and inhibiting demineralization, particularly in incipient carious lesions [20]. Clinical studies have demonstrated that fluoride varnish applications can substantially reduce caries incidence, with decreases of 30-45% in permanent teeth and 37% in primary teeth [21]. A systematic review further supports the efficacy of fluoride varnish, highlighting its superior effectiveness in high-risk populations, particularly among children with inadequate oral hygiene and frequent consumption of fermentable carbohydrates [22]. Fluoride varnish is typically applied biannually or quarterly based on a personalized evaluation of the child's caries risk. The ability of fluoride varnish to adhere to tooth surfaces for an extended period facilitates prolonged fluoride release, thus improving its efficacy in caries prevention [23]. This approach has demonstrated significant success in low-resource environments, primarily owing to its cost-effectiveness and ease of application, which does not necessitate specialized equipment or extensive professional training [24]. Despite its recognized benefits, compliance-related challenges remain a significant barrier, especially for populations with limited access to routine dental care. The consistent application and incorporation of fluoride varnish programs into school or community health initiatives may serve as effective strategies to address these challenges and enhance caries prevention strategies [10]. 

Pit and fissure sealants: Dental sealants are thin, protective coatings applied to the occlusal surfaces of posterior teeth. Their main function is to inhibit plaque accumulation in the deep pits and fissures, thereby significantly reducing the risk of dental caries [25]. There are two primary types of sealants: (a) Resin-based sealants exhibit superior retention and mechanical strength due to their capacity to establish micromechanical bonds with enamel. However, the implementation of these methods requires stringent moisture control, making the process especially susceptible to technical discrepancies [26]. (b) Glass ionomer sealants provide the significant advantage of fluoride release, facilitating the remineralization process and offering extended protection against dental caries. However, their retention rates are relatively inferior to those of resin-based sealants [27]. A systematic review demonstrated that salaries substantially reduce the risk of caries in permanent molars by 76% within two years of application, sustaining an effectiveness of 60% after four years [26]. A comprehensive community-based study demonstrated that children who received sealants had a 3.6-fold decrease in the risk of developing caries compared to their peers who did not receive sealant treatment [28]. This study's results highlight the substantial benefits of sealants in reducing caries incidence and improving long-term oral health. The American Dental Association (ADA) and the Centers for Disease Control and Prevention (CDC) recognize the substantial advantages of sealant application, endorsing its application on all permanent molars in children as a key preventive strategy [29]. Besides preventing caries, sealants significantly reduce costs by decreasing the need for restorative treatments, thereby enhancing overall oral health outcomes [29]. Despite the well-documented benefits of sealants, their widespread adoption remains hindered by several challenges. Research has identified that clinician reluctance, inadequate awareness, and logistical challenges present significant obstacles to the execution of school-based sealant programs [30]. Some practitioners have expressed apprehensions regarding the durability of sealants and the possibility of caries advancing undetected beneath the sealant material. Research indicates that sealed non-cavitated lesions demonstrate a diminished risk of progression relative to unsealed lesions, highlighting the safety and efficacy of sealant application [26]. Addressing these challenges requires increasing awareness among clinicians and the general public about the benefits and safety of sealants. Improving school-based sealant programs and integrating them into broader public health initiatives could significantly enhance accessibility, particularly for frequently underserved communities [30]. Moreover, instituting policies that incentivize dental practitioners to incorporate sealants into routine pediatric care can further promote their utilization, thus providing enhanced protection against caries in children [30].

Silver diamine fluoride (SDF): Silver diamine fluoride is a fluoride-based solution that is non-invasive and has received considerable attention for its efficacy in arresting caries progression. The operational mechanism combines the antibacterial properties of silver with the remineralizing effects of fluoride, effectively arresting caries progression while simultaneously strengthening both enamel and dentin [31,32]. The dual-action properties of SDF make it an essential component in minimally invasive dentistry, providing considerable benefits for pediatric patients and individuals with specific healthcare needs [33,32]. SDF provides substantial advantages for young children and individuals who struggle with conventional restorative therapies, especially those with behavioral or cognitive difficulties [34]. Clinical studies and systematic reviews have consistently demonstrated that silver diamine fluoride effectively arrests the progression of caries in 80-90% of treated lesions, positioning it as one of the most efficacious non-restorative strategies for caries management [34,32]. A systematic review and meta-analysis demonstrated that silver diamine fluoride markedly inhibits the progression of caries in primary teeth relative to other preventive methods [33]. The application process is uncomplicated, induces minimal discomfort, and requires only a short duration in the chair, rendering it particularly advantageous for pediatric patients who may exhibit less cooperation [34]. Furthermore, the cost-effectiveness of SDF has positioned it as a feasible solution for addressing early childhood caries in underserved communities, especially via community-based and public health initiatives [32]. Although SDF exhibits considerable clinical efficacy, a significant drawback is the enduring black discoloration of carious lesions. This factor often raises apprehensions among parents, particularly regarding aesthetic considerations [11]. A survey investigating parental acceptance of silver diamine fluoride indicated that 56% of parents harbored reservations regarding its use, especially in relation to anterior teeth [11]. However, when parents were informed of its ability to prevent further decay and reduce the need for more invasive treatments, 76% indicated a readiness to utilize SDF on posterior teeth [35,36].

An additional study demonstrated that parental acceptance improved following education on the advantages of silver diamine fluoride, particularly in cases where it served as an alternative to sedation or general anesthesia for children displaying significant anxiety or behavioral challenges [36]. The findings underscore the essential importance of comprehensive parental education and informed consent in enabling the successful execution of SDF [36]. To mitigate the aesthetic concerns associated with silver diamine fluoride, some clinicians have explored supplementary treatments, such as the Silver-Modified Atraumatic Restorative Technique (SMART), which is a significant approach that combines silver diamine fluoride with glass ionomer cement. Applying glass ionomer cement to seal the stained lesion improves aesthetic outcomes while facilitating fluoride release and enhancing the restoration's longevity [32]. Due to its simple application, cost-efficiency, and substantial evidence supporting its efficacy, silver diamine fluoride has garnered widespread endorsement from both national and international dental organizations as a principal approach for caries management [32]. The American Dental Association and the American Academy of Pediatric Dentistry (AAPD) endorse the integration of silver diamine fluoride into public health programs, particularly for high-risk pediatric populations [32]. School-based fluoride programs and community health initiatives have successfully incorporated silver diamine fluoride into preventive dental care, resulting in a significant reduction in caries rates among low-income and underserved populations [32]. Expanding insurance coverage for SDF applications and increasing professional training in their use could markedly enhance accessibility and facilitate their extensive incorporation into clinical practice [32]. The advantages and disadvantages of silver diamine fluoride are summarized in Table 1. 

**Table 1:** The advantages and disadvantages of silver diamine fluoride. SHCN: Special healthcare needs. References: [7,8,27-31].

Technique	Advantages	Disadvantages
Silver diamine fluoride	Arrests 80–90% of caries lesions	Causes permanent black staining of carious lesions
	Non-invasive and quick application	
	Suitable for SHCN and anxious patients	Low aesthetic acceptance (especially anterior teeth)
	Cost-effective	

Diet and sugar control: The dietary choices we make profoundly affect the prevention of dental caries; consistent consumption of fermentable carbohydrates stimulates acid production by oral bacteria, thereby facilitating enamel demineralization and the development of caries [37]. The World Health Organization (WHO) recommends restricting free sugar intake to a maximum of 10% of total daily energy consumption, with an additional suggestion to lower this to 5% for optimal oral health outcomes [38]. Studies have consistently demonstrated that reducing sugar intake significantly decreases the prevalence of dental caries across diverse populations. This highlights the significance of dietary modifications as an essential element of effective caries prevention strategies [38]. Public health initiatives, such as sugar taxation policies, have proven highly effective in reducing the consumption of sugar-sweetened beverages (SSBs), which is closely linked to the incidence of dental caries, obesity, and various metabolic disorders [39]. A study investigating the convergence of economics and epidemiology in India indicated that implementing a tax on sugar-sweetened beverages could prevent millions of cases of obesity and type 2 diabetes, as well as reduce the prevalence of sugar-related oral diseases [34]. Moreover, community education programs aimed at elucidating the cariogenic characteristics of certain foods have proven effective in promoting healthier dietary choices among children and their guardians [38].

Oral hygiene education: Consistent and effective oral hygiene practices are essential for preventing dental caries and promoting overall oral health. A meta-analysis study indicates that brushing twice daily with fluoridated toothpaste results in a 24% reduction in the incidence of dental caries compared to brushing once daily [40]. Fluoride toothpaste plays a crucial role in remineralizing enamel and neutralizing acids produced by plaque biofilms, underscoring its importance in daily oral hygiene routines. Initiatives incorporating supervised toothbrushing and organized oral health education have shown a significant improvement in adherence and oral hygiene outcomes, particularly among school-aged children [41]. A comparative study examining urban and rural schoolchildren indicated that participants in school-based toothbrushing programs exhibited significant improvements in oral hygiene, gingival health, and caries prevention compared to their non-participating peers [41]. The findings highlight the essential influence of parents and educators in encouraging proper brushing techniques and oral hygiene habits, both at home and in schools [41]. The incorporation of technology in oral health education, demonstrated through interactive mobile applications and gamification, has proven effective in engaging children and supporting the continuation of their oral hygiene practices. The utilization of these digital tools has demonstrated potential in improving motivation, compliance, and awareness concerning caries prevention in children [41].

Minimally Invasive Restorative Techniques 

Atraumatic restorative treatment (ART): Atraumatic restorative treatment is a non-invasive method designed to treat dental caries, utilizing minimal intervention techniques that avoid the need for using rotary instruments and local anesthesia. The procedure involves the meticulous removal of necrotic tissue using hand instruments, followed by the application of glass ionomer cement. This substance, recognized for its fluoride-releasing characteristics, enhances adhesion and provides long-term protection against secondary caries formation [40]. ART has gained significant recognition, particularly in low-resource settings where access to conventional restorative procedures, including rotary instruments and anesthesia, is limited [41]. The approach's simplicity, efficacy, and cost-effectiveness make it an essential strategy in community and school oral health programs [42]. The efficacy of ART has been extensively documented in both primary and permanent dentition, with studies indicating survival rates comparable to conventional restorative techniques. A meta-analysis revealed that ART restorations in primary teeth exhibited survival rates ranging from 75% to 85% over a three-year period, underscoring their reliability as an alternative to amalgam or composite restorations [42]. A clinical study conducted over three years in Thailand demonstrated that single-surface cavities in permanent teeth attained a success rate of 93%, whereas multiple-surface lesions achieved a success rate of 80% [43]. The findings underscore the efficacy of ART as a viable restorative solution, particularly in regions with limited access to advanced dental care [43]. However, factors such as patient compliance, the operator's expertise, and environmental conditions, including moisture control during placement, are acknowledged as critical elements influencing the longevity of restorations [44]. A significant advantage of ART is its cost-effectiveness and the ease of its integration into public health initiatives [45]. Numerous dental programs, both in educational institutions and community environments, have successfully implemented the ART approach, thereby improving access to restorative care for frequently underserved populations [46]. A systematic review examining socioeconomic disparities and dental caries revealed that ART-based programs significantly improved oral health outcomes in disadvantaged populations. This highlights its potential as a scalable and sustainable method for addressing global oral health disparities [45]. Although ART provides numerous advantages, it also entails specific limitations that influence its wider acceptance. A primary challenge is the decreased success rate noted in multi-surface lesions, where increased mechanical stress on restorations can lead to premature failure [44]. Furthermore, while glass ionomer cement (GIC) provides the advantage of fluoride release that contributes to ongoing caries prevention, it lacks the wear resistance found in composite or amalgam restorations. This limitation makes it more susceptible to premature deterioration, particularly in individuals who apply considerable masticatory forces [44]. ART is particularly sensitive to technique, requiring optimal moisture control during application to ensure optimal bonding and longevity of restorations [44]. To enhance the efficacy of ART, future research and policy initiatives must focus on improving material properties, expanding training programs, and integrating ART into comprehensive healthcare coverage efforts. Recent advancements in glass ionomer cement formulations, especially the development of reinforced materials with improved wear resistance and durability, may significantly increase the success rates of atraumatic restorative treatment. Furthermore, expanding training opportunities for dental professionals, community health workers, and school-based oral health personnel may facilitate the expansion of ART programs, thereby enhancing accessibility [46]. The advantages and disadvantages of atraumatic restorative treatment are summarized in Table 2.

**Table 2:** The advantages and disadvantages of atraumatic restorative treatment References: [9,41-46].

Technique	Advantages	Disadvantages
Atraumatic restorative treatment	Does not require rotary instruments or anesthesia	Reduced success in multi-surface cavities
	Suitable for low-resource settings	
	High survival in single-surface lesions	Technique-sensitive with moisture control

Hall technique: The Hall technique is a non-invasive approach for treating dental caries, involving the placement of a preformed metal crown (PMC) on a decayed primary tooth, typically a molar. This procedure is unique as it does not necessitate the removal of decayed tissue, the administration of local anesthesia, or any drilling for tooth preparation [47]. This technique efficiently seals cavities, thereby obstructing the nutrient supply to deleterious bacteria responsible for dental caries. Consequently, it inhibits caries progression and prevents further deterioration of the affected tooth [48]. The Hall technique distinguishes itself from conventional restorations by removing carious dentin without requiring cavity preparation. This technique effectively prevents the progression of dental lesions while avoiding the need for mechanical excavation. It is particularly beneficial for children who suffer from dental anxiety or exhibit behavioral difficulties [48,49]. Numerous studies have demonstrated remarkable clinical success rates, with a 97% survival rate over five years. This performance significantly exceeds that of teeth treated with pulpotomy and composite restorations, especially in terms of durability and caries prevention [49]. A randomized controlled trial (RCT) demonstrated that Hall crowns resulted in decreased post-operative pain and discomfort, thereby improving patient compliance and overall satisfaction with the treatment [50]. A systematic review from the Cochrane Database has shown that Hall crowns provide benefits compared to conventional restorations, including enhanced durability, reduced failure rates, and increased patient acceptability. Moreover, they correlate with a reduced occurrence of secondary caries and a reduced need for retreatment [51]. The increased acceptance rate among children and their parents, especially those with special healthcare needs, signifies a significant advantage. Studies indicate that 87% of parents prefer the Hall technique, due to its minimally invasive approach, reduced treatment time, and lower discomfort levels [52]. However, concerns regarding the aesthetic appearance of stainless steel crowns, especially on visible posterior teeth, remain a challenge [52]. Parental acceptance significantly increases when informed about the superior success rates and enduring benefits of preventing further decay [53]. This technique offers multiple advantages, including the elimination of drilling, anesthesia, and multiple follow-up appointments. It reduces dental fear and anxiety while providing long-term protection against caries with minimal maintenance [49,51]. The efficient application process improves its appropriateness for pediatric patients who may be uncooperative or have special healthcare needs [50]. However, it is essential to consider challenges such as aesthetic factors, constraints in case selection, and transient alterations in occlusion [52,53]. While occlusal adaptation generally resolves within several weeks, this technique is most efficacious for primary molars that are not pulpally compromised. In instances of considerable structural damage, alternative interventions may be required [51,53]. Moreover, the training and exposure of clinicians remain significant challenges, underscoring the need for improved education and practical experience [53]. Future research should explore the application of tooth-colored zirconia crowns to improve aesthetic results and compare Hall crowns with novel bioactive restorative materials to optimize the management of pediatric caries [53]. The advantages and disadvantages of the Hall technique are summarized in Table 3.

**Table 3:** The advantages and disadvantages of Hall technique References: [48-53].

Technique	Advantages	Disadvantages
Hall technique	High success rate (up to 97%)	Aesthetic concerns due to metal crowns
	Avoids drilling, anesthesia, and multiple visits	
	High parental acceptance post-education	Not suitable for pulpally involved teeth

Resin infiltration: Resin infiltration represents a minimally invasive approach aimed at managing early enamel caries lesions and white spot lesions, eliminating the necessity for mechanical preparation or drilling [54]. This approach utilizes a low-viscosity resin that penetrates demineralized enamel, filling porous structures and stabilizing the lesion, thereby effectively preventing further progression of caries [55]. Originally designed for the management of proximal caries, resin infiltration has achieved significant clinical acceptance due to its efficacy in treating non-cavitated lesions, especially among pediatric and orthodontic patients [54]. The procedure begins with the use of an etching agent, commonly a 15% hydrochloric acid solution, which effectively eliminates the hypermineralized enamel surface layer. This action facilitates enhanced resin penetration into the underlying structures [55]. Following this, a low-viscosity resin infiltrant is applied and cured using light polymerization, effectively sealing the lesion and inhibiting further demineralization [56]. Clinical studies emphasize its efficacy in managing early white spot lesions post-orthodontic treatment, demonstrating that it not only arrests the progression of these lesions but also enhances aesthetic outcomes by integrating the lesion with the adjacent healthy enamel [56]. Due to its non-invasive characteristics, resin infiltration has become a preferred treatment option for pediatric patients, effectively maintaining enamel integrity and reducing discomfort for the patient [54]. Studies have shown that it is highly effective in stabilizing 80-90% of non-cavitated proximal lesions over a two-year period, particularly among individuals who are at high risk for caries [57]. A systematic review found that resin infiltration is superior to fluoride varnish in arresting the progression of lesions, particularly in the case of early-stage carious lesions where demineralization is confined to the enamel [57]. Furthermore, it proves to be highly effective in reducing lesion contrast, making it an ideal choice for the aesthetic management of post-orthodontic white spot lesions [56]. One of the significant advantages of resin infiltration is that it eliminates the need for drilling, thereby preserving healthy enamel and decreasing patient discomfort [57]. The technique provides both immediate and long-term advantages, providing immediate lesion stabilization while maintaining long-term effectiveness in preventing lesion recurrence [55]. Moreover, resin infiltration reduces reliance on fluoride-based therapies by physically occluding the porosities within demineralized enamel, effectively arresting lesion progression and providing a definitive approach for managing early caries [57].

While resin infiltration offers several benefits, it is important to note that the technique is sensitive and demands careful moisture management during its application. Even slight contamination from saliva can compromise the penetration of the resin and the strength of the bond, ultimately affecting its durability over time [58]. Furthermore, this technique demonstrates optimal effectiveness for non-cavitated enamel lesions; however, its efficacy decreases when cavitation progresses into the dentin, thereby requiring different restorative strategies for more advanced lesions [57]. Additional factors that can affect its success include patient compliance and oral hygiene habits, as diets high in sugar and inadequate oral care increase the risk of lesion recurrence, even after initial treatment [57]. The cost is an important factor to consider, as resin infiltration may involve a higher initial expense compared to fluoride varnish applications. Nevertheless, the long-term advantages, such as enhanced aesthetics and a decreased necessity for future restorations, frequently validate the investment [58]. In comparison, fluoride varnish promotes remineralization but requires ongoing fluoride exposure, while resin infiltration effectively obstructs lesion progression, thereby arresting further demineralization [57]. Sealants effectively establish a protective barrier against cariogenic biofilm; however, they do not penetrate or reinforce lesion structures as resin infiltration does, which also provides enhanced aesthetic outcomes [56]. Similarly, silver diamine fluoride is highly effective in arresting dental caries; however, its tendency to cause permanent black staining raises considerable aesthetic issues. Consequently, resin infiltration emerges as a preferable alternative for anterior teeth and patients who prioritize aesthetics [58]. Nonetheless, careful case selection remains critical, as resin infiltration is most effective in managing non-cavitated lesions and requires meticulous execution of the technique [57]. Future advancements in resin formulations are anticipated to incorporate bioactive components to enhance remineralization potential, reinforcing its significance as a promising approach in minimally invasive dentistry [58]. The advantages and disadvantages of resin infiltration are summarized in Table 4.

**Table 4:** The advantages and disadvantages of resin infiltration References [54-58].

Technique	Advantages	Disadvantages
Resin infiltration	Effective in non-cavitated lesions	Technique-sensitive (moisture control)
	Enhances esthetics (white spot masking)	
	Physically seals porosities, reducing the need for fluoride	Not suitable for cavitated or dentin lesions
		Cost may be higher

Bioactive materials: The advancement of bioactive restorative materials has significantly transformed minimally invasive dentistry. These materials enhance the durability, efficacy, and biological compatibility of dental restorations. They play a crucial role in promoting remineralization, facilitating pulp healing, and improving adhesion to tooth structures, which in turn reduces the necessity for conventional restorative procedures [59]. Among these materials, glass ionomer cement is commonly employed in pediatric and minimally invasive dentistry applications. Its fluoride-releasing properties, ability to chemically bond to dentin, and high biocompatibility contribute to its effectiveness in caries prevention and enamel remineralization [60]. Research has shown that high-viscosity glass ionomer cement restorations have survival rates similar to those of composite restorations in primary teeth, highlighting its potential as a cost-effective option in pediatric dentistry [61]. Nonetheless, the reduced mechanical strength and wear resistance of GIC limit its application in high-stress regions like molars. This limitation has led to the development of nano-sized fillers and bioactive nanoceramics aimed at improving their durability and functionality [62]. In contrast, materials based on calcium silicate, including Biodentine and TheraCal LC, have been recognized as more effective options due to their strong bonding with dentin, bioactive properties, and capacity to facilitate dentin regeneration, especially in cases of deep carious lesions and indirect pulp capping procedures [63]. Biodentine has demonstrated remarkable mechanical properties and the ability to promote pulp healing, surpassing conventional glass ionomer cements in terms of sealing effectiveness, microleakage prevention, and resistance to bacterial penetration. This contributes to the long-term success of minimally invasive dentistry restorations [64]. Similarly, TheraCal LC, a light-cured resin-modified calcium silicate, exhibits excellent moisture tolerance and biocompatibility, establishing it as a dependable pulp-protective liner to be used beneath restorations [65]. The incorporation of bioactive materials in minimally invasive dentistry presents notable benefits, such as the continuous release of fluoride, which supports the long-term remineralization of enamel and dentin, thereby decreasing the likelihood of secondary caries [60]. The materials in question demonstrate improved bonding and sealing capabilities, which effectively reduce microleakage and bacterial infiltration. This, in turn, contributes to greater longevity of restorations and enhances clinical outcomes [63]. Moreover, their excellent biocompatibility makes them especially appropriate for pediatric patients, providing safe and effective treatment alternatives that adhere to the principles of minimally invasive dentistry, which emphasize the preservation of tooth structure and the reduction of patient discomfort [59]. Furthermore, these materials play a significant role in enhancing aesthetics, especially in the field of pediatric dentistry, where modified resin-based bioactive materials offer a more visually appealing alternative to conventional glass ionomer cement [62]. The decrease in the necessity for invasive drilling methods corresponds with MID principles, facilitating the conservation of healthy tooth structure and reducing patient discomfort [59]. While bioactive restorative materials offer a range of benefits, various limitations impede their broader implementation in clinical settings. A significant challenge lies in the mechanical limitations of glass ionomer cement, which tends to fracture in load-bearing posterior teeth, thereby diminishing its appropriateness for high-stress areas [61]. Moreover, materials such as Biodentine demonstrate extended setting times, which may interfere with the efficiency of clinical workflows and extend the duration of treatment [64]. The increased expense of bioactive materials in comparison to conventional options limits their availability, especially in low-resource environments where cost-effectiveness is a significant issue [62]. Furthermore, a significant number of these materials, particularly those that are resin-modified, demand optimal moisture control during their application. This characteristic renders them particularly sensitive to technique and requires a high level of clinical expertise to achieve the best possible results [63]. Future advancements in modifications utilizing nanotechnology are focused on enhancing the strength, wear resistance, and fluoride-releasing capabilities of glass ionomer cement by integrating nanofillers and bioactive nanoceramics [62]. Innovative bioactive composites that integrate calcium phosphate nanoparticles are currently being designed to improve remineralization, all while preserving both aesthetic and mechanical strength [64]. Research is currently investigating hybrid materials that combine glass ionomer cements, resin composites, and calcium silicate cements. The aim is to enhance adhesion, bioactivity, and durability [66]. The development of bioactive restorative materials represents a significant advancement in minimally invasive dentistry, contributing to improved caries management, remineralization, and the preservation of tooth structure [59]. Although glass ionomer cement continues to be a fundamental choice due to its ability to release fluoride, calcium silicate-based materials such as Biodentine and TheraCal LC offer enhanced mechanical properties and advantages for pulp healing [63,64]. Despite the ongoing challenges related to cost and mechanical limitations, current research in nanotechnology and bioactive modifications is progressively improving clinical effectiveness [62]. The advantages and disadvantages of bioactive restorative materials are summarized in Table 5.

**Table 5:** The advantages and disadvantages of bioactive restorative materials GIC: Glass ionomer cement. References:  [59-66].

Technique	Advantages	Disadvantages
Bioactive restorative materials	Fluoride-releasing (GIC)	GIC has low wear resistance
	Promotes remineralization and pulp healing	
	Suitable for pediatric use	Biodentine has a longer setting time
	Biocompatible	Higher cost than conventional materials

Applications of Minimally Invasive Dentistry in Special Populations

Children with special healthcare needs often face significant challenges in accessing standard dental care due to behavioral, medical, and cognitive impairments that impede their capacity to tolerate conventional restorative treatments [67]. In many cases, dental procedures necessitating general anesthesia (GA) become imperative, increasing both the financial burden and the risk of complications [68]. Minimally invasive dentistry offers an alternative approach that reduces the need for general anesthesia and sedation by implementing non-invasive and patient-centered caries management techniques. Silver diamine fluoride is an exceptionally effective, minimally invasive dentistry approach for patients with special healthcare needs, as it arrests caries progression through a simple, painless application, thereby eliminating the need for drilling or injections [69]. The Hall technique involves invasive procedures and improving overall treatment compliance [52]. However, parental concerns regarding the aesthetic effects of SDF-induced discoloration and clinicians' hesitance to utilize unconventional restorative techniques remain significant barriers to the implementation of MID in this population placement of preformed metal crowns on decayed primary molars without removing the decay, demonstrating efficacy in treating uncooperative patients with special healthcare needs, thus reducing the need for [70]. Furthermore, many dental professionals lack the requisite specialized training in behavior management techniques essential for the effective application of MID in patients with special healthcare needs [71]. Improving training programs and increasing awareness among clinicians and caregivers can alleviate these challenges [72].

Emerging Technologies and Future Directions

Laser-assisted caries removal: Laser-assisted caries removal signifies a notable progression in minimally invasive dentistry, enhancing the precision and comfort of cavity preparation while preserving the integrity of healthy tooth structure [73]. Particularly, Erbium: Yttrium-Aluminum-Garnet (Er:YAG) and Erbium, Chromium: Yttrium-Scandium Gallium-Garnet (Er,Cr:YSGG) lasers have gained prominence among laser types due to their selective tissue ablation, bactericidal effects, and ability to create smear layer-free surfaces, which improve bond strength and the durability of restorations [74]. Research indicates that cavities treated with lasers exhibit reduced microleakage, consequently enhancing the durability of restorations [75]. Laser-assisted caries removal significantly diminishes pain perception, thereby reducing dependence on local anesthesia. This is particularly beneficial for pediatric patients and individuals with specialized healthcare needs [76]. Moreover, lasers diminish vibrations and mechanical pressure, which frequently exacerbate dental anxiety in children. However, challenges to widespread implementation persist, including the substantial costs of laser technology, prolonged treatment duration, and the necessity for clinicians to receive specialized training [77]. The advantages and disadvantages of laser-assisted caries removal are summarized in Table 6.

**Table 6:** The advantages and disadvantages of laser-assisted caries removal References: [73-77].

Technique	Advantages	Disadvantages
Laser-assisted caries removal	Selective tissue removal	High equipment cost
	Reduces pain and the need for anesthesia	
	Bactericidal effects	Requires clinician training
	Improves the bonding surface	Time-consuming compared to rotary methods

Air abrasion technology: Air abrasion is a minimally invasive alternative to conventional drilling, utilizing fine aluminum oxide particles to selectively remove carious tissue without the application of heat, pressure, or vibration [78]. This technique is especially efficacious for non-cavitated lesions and small decayed areas, offering a conservative approach that preserves greater amounts of intact enamel and dentin [79]. Clinical studies indicate that air abrasion markedly improves the dental experience for children by alleviating patient anxiety and discomfort, in contrast to rotary instruments [80]. Moreover, the technique facilitates the accurate removal of caries, facilitating the adhesion of bonding agents and improving the retention of restorations [78]. Nonetheless, air abrasion possesses limitations, including the necessity for optimal moisture control, the difficulty in removing deep caries, and higher operational expenses relative to conventional techniques [78]. Further investigation is necessary to evaluate its long-term effectiveness [78]. The advantages and disadvantages of air abrasion are summarized in Table 7.

**Table 7:** The advantages and disadvantages of air abrasion References: [78-80].

Technique	Advantages	Disadvantages
Air abrasion	Preserves sound tooth structure	Limited to small, accessible lesions
	No vibration or heat	
	Comfortable for pediatric patients	Requires strict moisture control
		Higher operating cost

Teledentistry and digital innovations: Teledentistry has revolutionized the delivery of pediatric dental care, particularly in underserved and remote regions [81]. The rapid adoption of the COVID-19 pandemic facilitated remote consultations, triage, follow up, and oral health education through digital platforms [82]. A systematic review indicated that teledentistry is approximately 80% as effective as in person visits for diagnosing early childhood caries and monitoring oral hygiene compliance [83]. The integration of artificial intelligence (AI) in teledentistry has enhanced diagnostic precision and treatment planning by facilitating caries detection, radiograph analysis, and caries risk assessment [84]. Its widespread application is hindered, however, by barriers such as restricted insurance coverage, technical discrepancies, and regulatory obstacles [81].

Artificial intelligence in pediatric dentistry: The incorporation of artificial intelligence into pediatric minimally invasive dentistry is increasingly common, demonstrating significant potential to enhance diagnostic accuracy and treatment strategy. AI algorithms, such as convolutional neural networks (CNNs), exhibit an impressive 95% sensitivity in detecting early carious lesions on pediatric radiographs. This performance exceeds that of traditional visual tactile methods and reduces the subjectivity frequently linked to diagnostics [85]. A 2023 study indicated that AI-driven tools like DeepCaries™ improved caries detection rates by 25% in primary teeth relative to conventional methods, enabling earlier intervention and fostering minimally invasive care [86]. Artificial intelligence improves the formulation of personalized treatment strategies by evaluating the risk of caries progression through the analysis of demographic, dietary, and microbiological data. A 2022 model exhibited an exceptional 89% accuracy in identifying pediatric patients at elevated risk [87]. In teledentistry, artificial intelligence-enhanced platforms evaluate smartphone images to prioritize care for children in underserved regions. This novel method has resulted in a 35% decrease in untreated caries within rural populations via the implementation of remote silver diamine fluoride treatments [88] Nonetheless, challenges remain, notably the inadequate diversity in training datasets, which may lead to biased AI performance among different ethnic and socioeconomic groups, as emphasized in a 2023 analysis of pediatric MID studies [89]. Ethical considerations concerning data privacy and algorithmic transparency necessitate the establishment of rigorous regulatory frameworks to guarantee the effective and equitable operation of AI tools [90]. Future research should emphasize multicenter trials to confirm the long-term efficacy of AI, incorporating caregiver feedback to enhance user experience [91]. The advantages and disadvantages of teledentistry and AI are summarized in Table 8.

**Table 8:** The advantages and disadvantages of teledentistry and AI References: [81-91].

Technique	Advantages	Disadvantages
Teledentistry + AI	Enhances early diagnosis and follow-up in remote areas	Requires internet and tech access
	Useful in caries risk prediction and patient education	Regulatory and insurance challenges
		Accuracy varies by model

## Conclusions

Minimally invasive dentistry is revolutionizing pediatric dental care by providing biocompatible, cost-effective alternatives to conventional invasive procedures. This study demonstrates the efficacy of minimally invasive dentistry techniques such as atraumatic restorative treatment, the Hall technique, resin infiltration, bioactive materials, laser-assisted caries removal, air abrasion, teledentistry and artificial intelligence in conserving tooth structure, thereby alleviating dental anxiety and enhancing treatment outcomes. Nonetheless, challenges such as insufficient long-term clinical data, high costs, technological limitations, and regulatory discrepancies impede widespread acceptance. Subsequent research projects should primarily consist of multi-center longitudinal studies to assess the impacts on oral health, cost-effectiveness, and durability of MID techniques. Initiating public health campaigns, increasing insurance reimbursement, and incorporating MID into dental curricula facilitate enhanced acceptance. The clinical efficacy of MID will continue to be enhanced by advancements in digital health technologies, bioactive materials, and artificial intelligence-driven diagnostics. Through research, policy reform, and clinician education, MID can continue to evolve as the gold standard for pediatric caries management, ensuring minimally invasive, efficient, and sustainable dental care for children worldwide.
